# Supplementary material for: A Genetic Code Expansion‐Derived Molecular Beacon for the Detection of Intracellular Amyloid‐β Peptide Generation
Source: Angew Chem Int Ed Engl. 2020 Dec 15;60(8):3934–9. doi: 10.1002/anie.202010703 (PMC7898502; doi:10.1002/anie.202010703)
Supplement: Supplementary file 1 — Supplementary [file ANIE-60-3934-s001.pdf]

Angewandte Chemie
Eine Zeitschrift der Gesellschaft Deutscher Chemiker

Supporting Information

A Genetic Code Expansion-Derived Molecular Beacon for the Detection of Intracellular Amyloid-β Peptide Generation

*Khomkrit Sappakhaw, Krittapas Jantarug, Sarah A. Slavoff, Nipan Israsena, and Chayasith Uttamapinant**

anie_202010703_sm_miscellaneous_information.pdf

Content:

Figure S1. APP expression via genetic code expansion.

Figure S2. More fields of view from genetic code expansion labeling of different amyloid precursor protein (APP) amber mutants.

Figure S3. Fluorescent labeling of APP(BCNK) variants with tetrazine-cy5.

Figure S4. Minimal labeling background from translation readthrough and non-specific sticking of fluorophores.

Figure S5. Detection of amyloid-β species via antibody and amyloid plaque-specific dye.

Figure S6. Exogenous Aβ expression has different localization patterns from Aβ natively processed from APP.

Figure S7. Western blot analysis of APP(H609BCNK)-HaloTag with different antibodies.

Figure S8. Mass and liquid chromatography characterizations of QSY21-chloroalkane.

Figure S9. Quantification of FRET-based quenching efficiency of cy5 on APP(H609BCNK)-HaloTag by QSY21-chloroalkane.

Figure S10. HaloTag on cy5-labeled APP is needed for effective quenching by QSY21-chloroalkane.

Methods

Reference

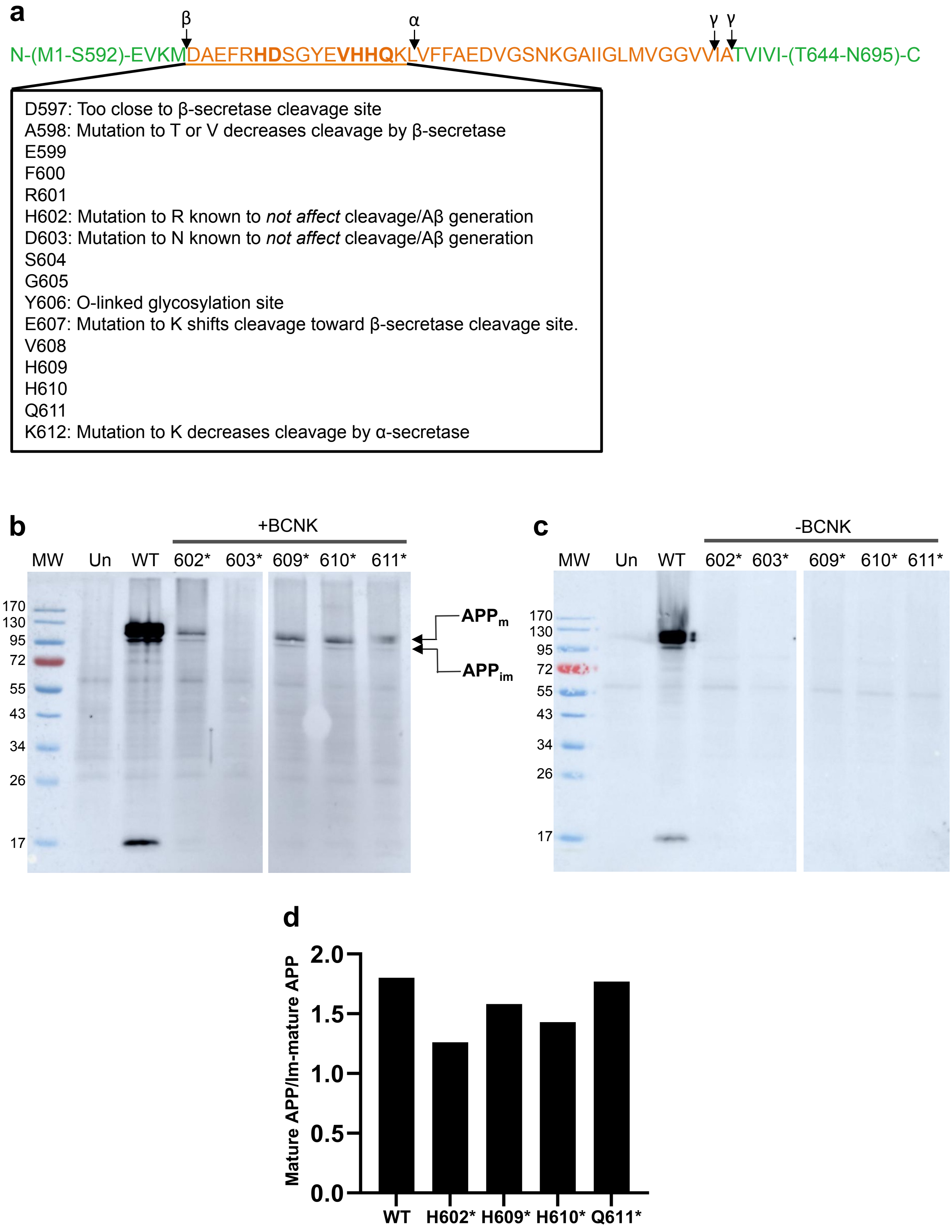

Figure S1. APP expression via genetic code expansion. (**a**) Criteria for amino acid position selection to be mutated to an amber stop codon for genetic code expansion. (**b,c**) Confirming BCNK-dependent expression of APP. HEK293 cells were transfected with transgenes encoding various APP(TAG)-myc mutants plasmids, incubated 45 hours in the presence or absence of 60 µM BCNK and analysed on western blots using anti-α-myc antibody. TAG mutation is represented by *. APP_m and APP_{im} indicate bands corresponding to mature-APP and immature-APP, respectively. (**d**) Quantification of intensity ratio between mature-APP and immature-APP of various APP(TAG) mutants.

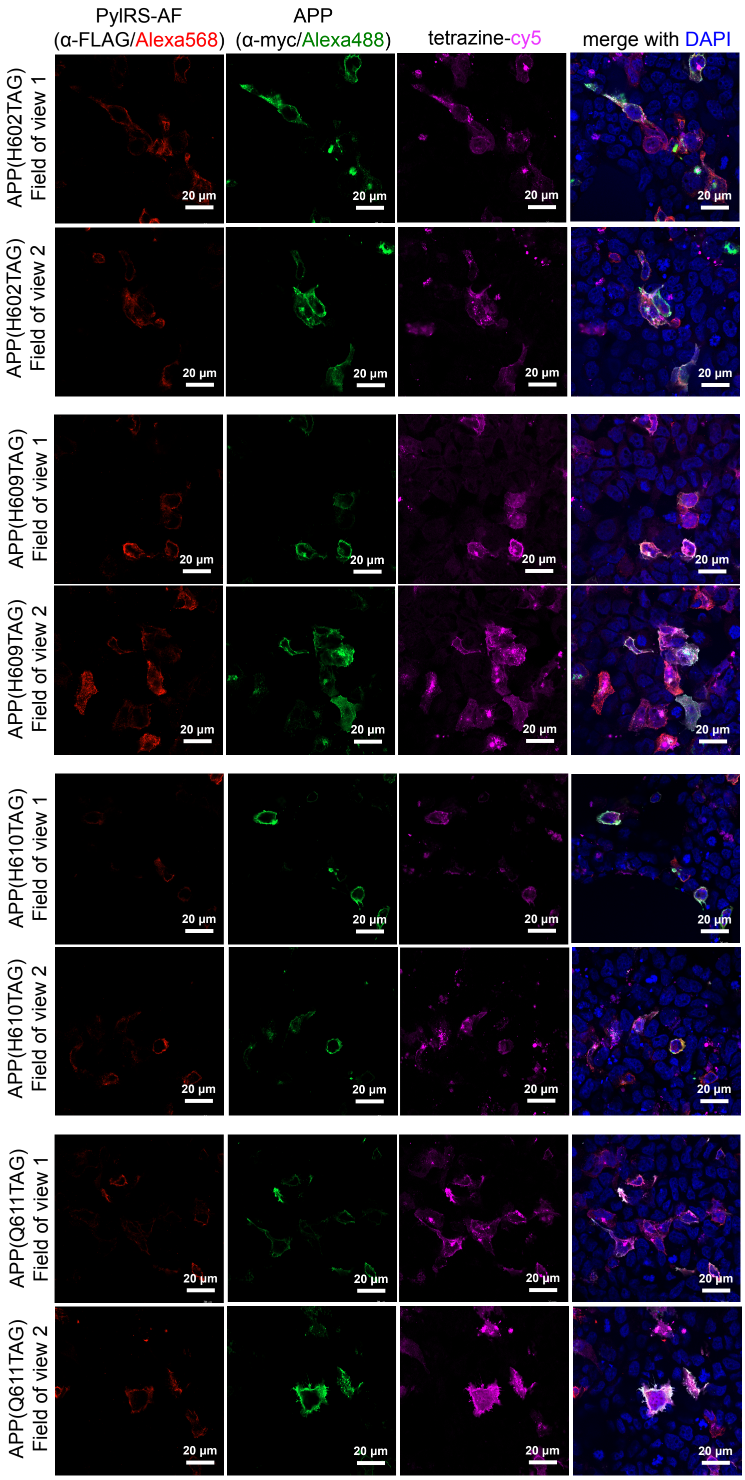

Figure S2. More fields of view from genetic code expansion labeling of different amyloid precursor protein (APP) amber mutants. HEK293 cells were transfected with transgenes encoding APP(TAG)-myc mutants, PylRS-AF and Pyl-tRNA$_{CUA}$, and incubated with 60 µM BCNK for 45 hours. Cells were then labeled live with 1.2 µM tetrazine-cy5, fixed and counterstained with anti-myc for APP expression, and anti-FLAG for PylRS-AF expression.

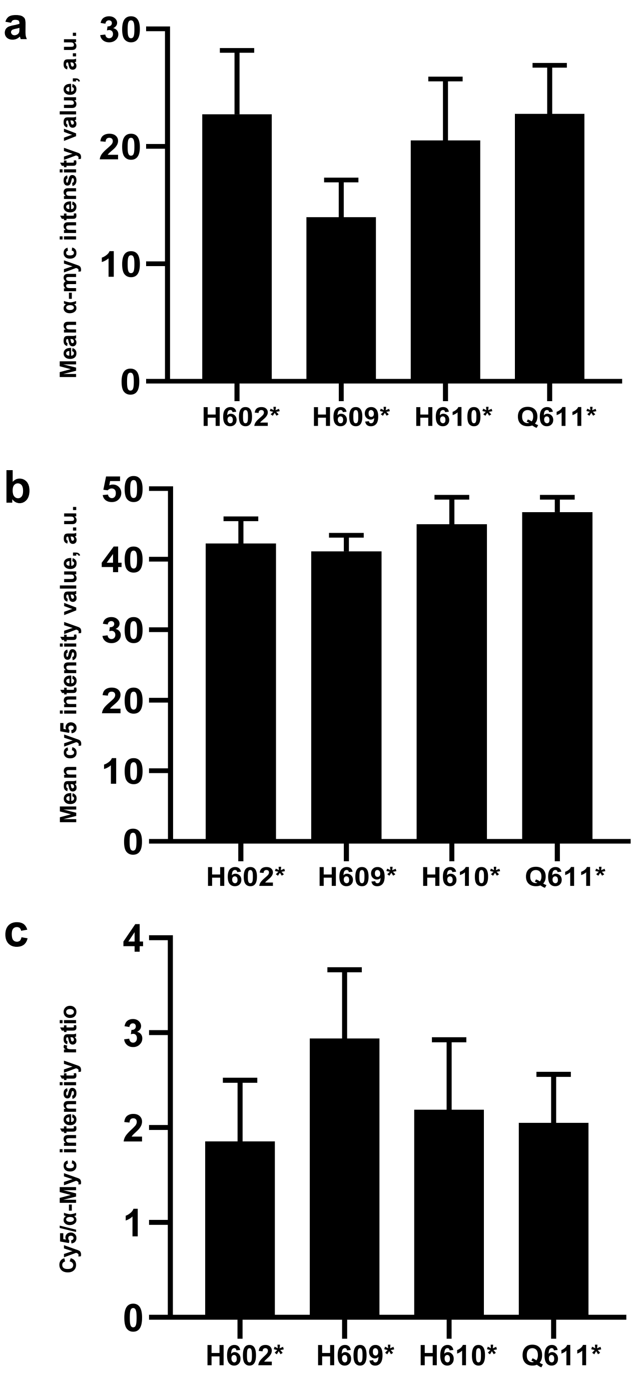

Figure S3. Fluorescent labeling of APP(BCNK) variants with tetrazine-cy5. (a) α-myc/Alexa488, **(b)** tetrazine-cy5 and **(c)** intensity ratio between tetrazine-cy5 and α-myc/Alexa488**.** Values shown are mean ± SEM. Each condition represents over 50 cells analyzed.

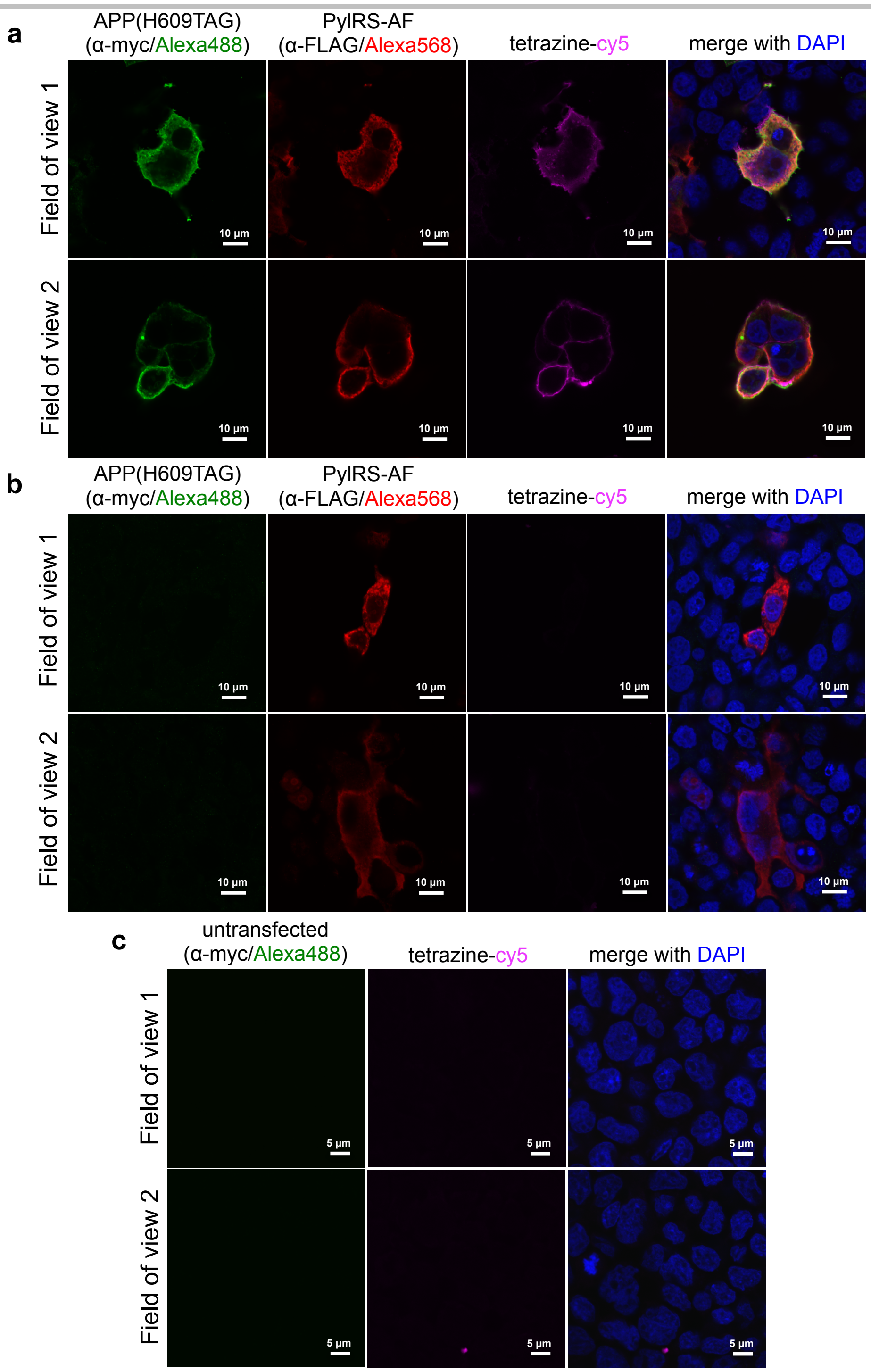

Figure S4. Minimal labeling background from translation readthrough and non-specific sticking of fluorophores. (a,b) Comparison of specific labeling signal on amber-containing APP vs labeling background from amber stop codon readthrough. HEK293 cells were transfected with (a) transgenes encoding APP(H609TAG)-myc, PylRS-AF and Pyl-tRNA$_{CUA}$, or (b) only PylRS-AF and Pyl-tRNA$_{CUA}$. Cells were incubated with 60 µM BCNK for 45 hours, labeled live with 1.2 µM tetrazine-cy5 at 4 °C for 30 minutes, immediately fixed, and immunostained with anti-myc for APP expression, and anti-FLAG for PylRS-AF expression, **(c) Minimal non-specific sticking of tetrazine-cy5 on cells.** Untransfected HEK293 cells were grown in the presence of 60 µM BCNK for 45 hours, and incubated with 1.2 µM tetrazine-cy5 as in (a) before fixing and imaging.

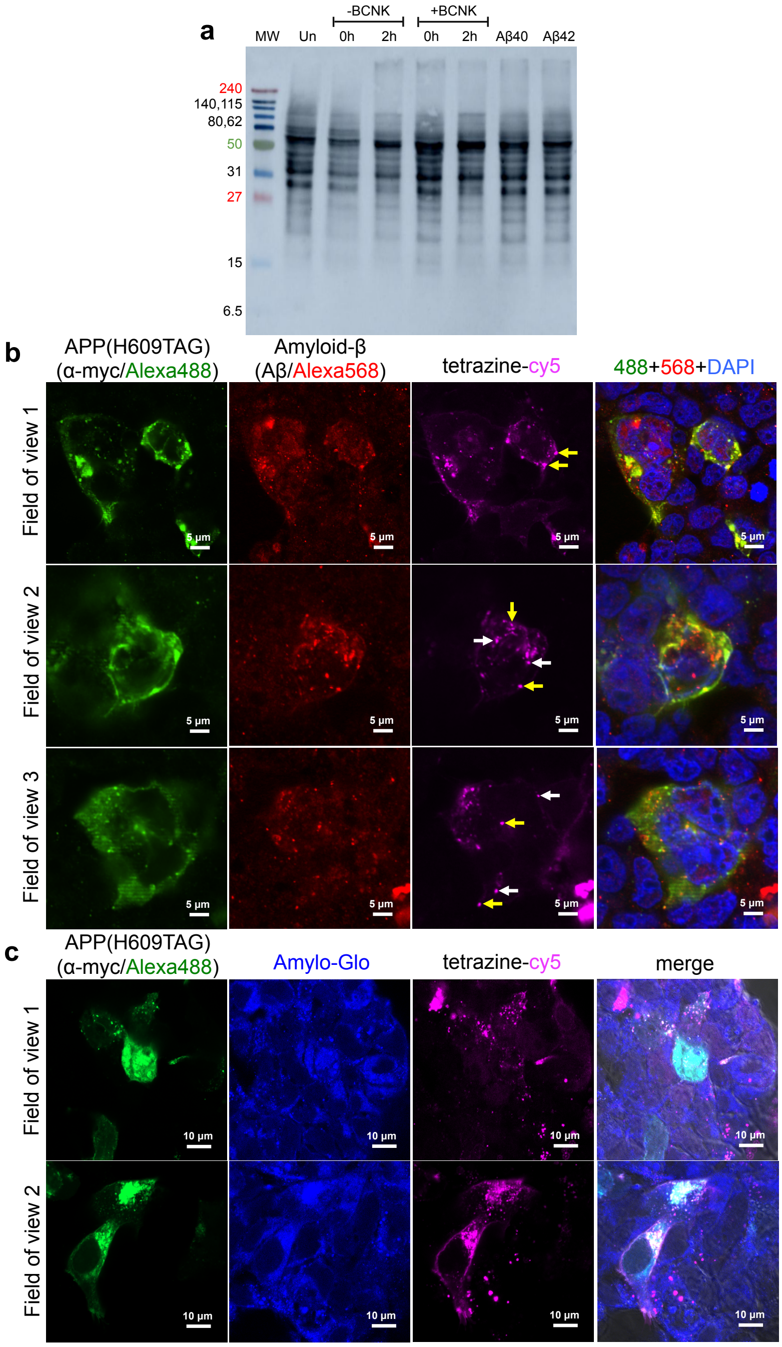

Figure S5. Detection of amyloid-β species via antibody and amyloid plaque-specific dye. (a) High background upon performing Western blotting with anti-amyloid antibody. HEK293 expressing APP(H609BCNK)-myc were lysed with RIPA buffer either immediately (0h) or 2-hour to allow APP processing (2h), before analysis by Western Blotting with anti-Aβ antibody (Merck AB5078P). Controls were shown with untransfected (Un) cells, and HEK293 cells expressing exogenous Aβ40 and Aβ42. **(b) Specific labeling could be seen upon immunofluorescence staining and imaging with anti-amyloid antibody.** HEK293 expressing APP(H609BCNK)-myc were labeled live with 1.2 µM tetrazine-cy5 at 4 °C for 30 minutes and further grown for additional 2 hours at 37 °C to permit APP internalization and processing before fixation. Cells were counterstained with anti-myc for APP expression, and anti-Aβ for detection of amyloid-β. Yellow arrows indicate intact APP (coincident myc-staining and cy5 label) that was stained with anti-Aβ. White arrows indicate liberated Aβ (standalone cy5 signal) that was stained with anti-Aβ. **(c) Plaque-specific Amylo-Glo failed to specifically label intracellular APP and Aβ.** Amylo-glo staining was performed per manufacturer's instruction.

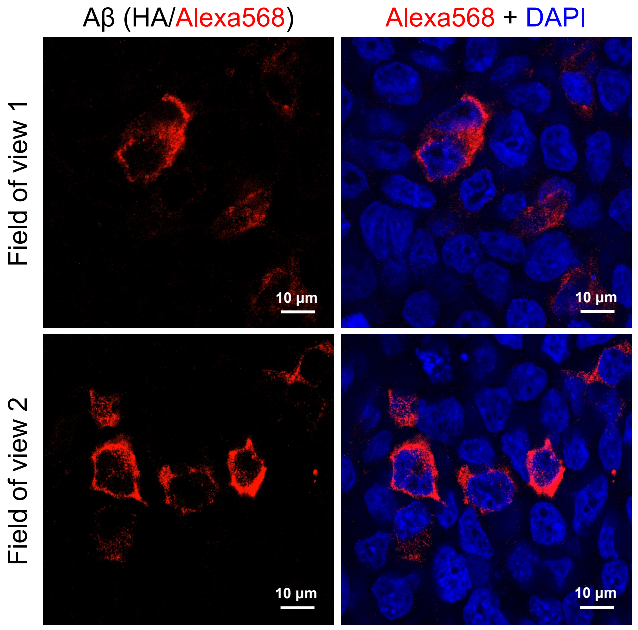

Figure S6. Exogenous Aβ expression has different localization patterns from Aβ natively processed from APP. Here, HEK293 cells were transfected with HA-Aβ40 plasmid, grown for 45 hours, fixed and analyzed by anti-HA antibody (HA/Alexa568).

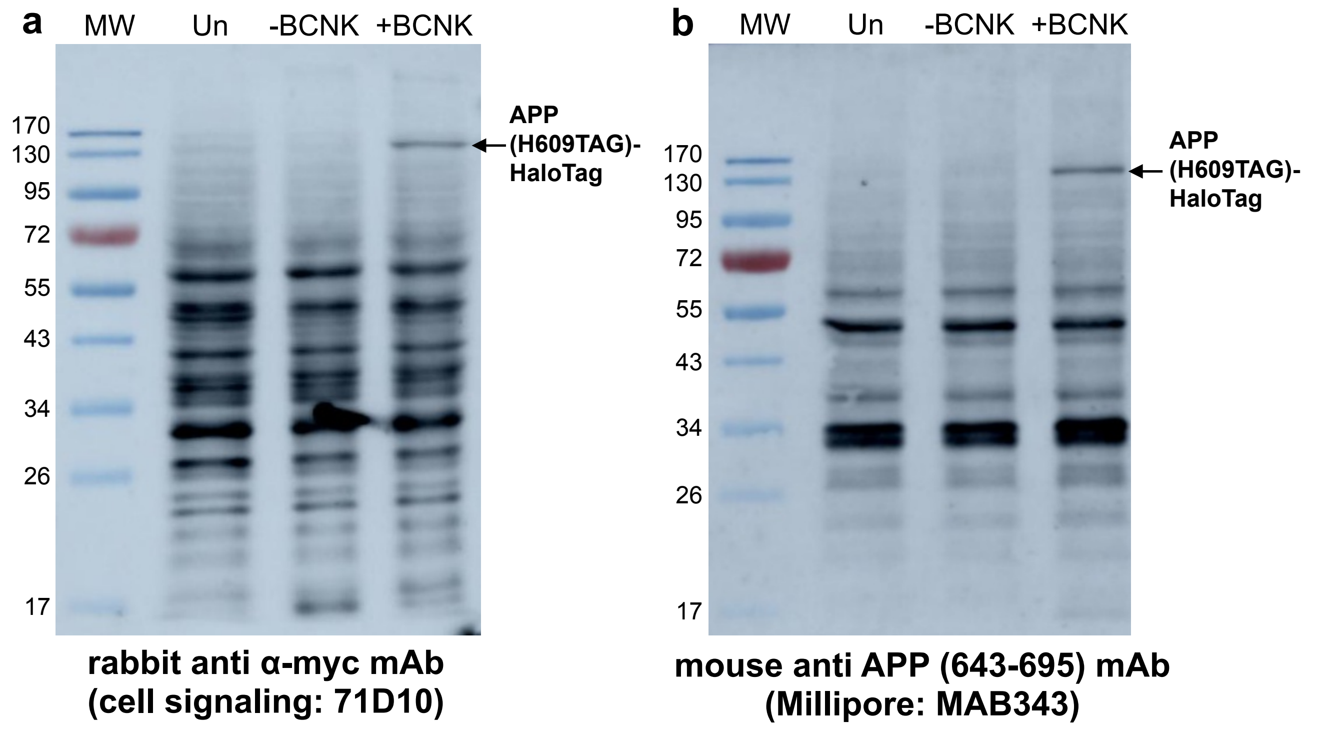

Figure S7. Western blot analysis of APP(H609BCNK)-HaloTag with different antibodies. HEK293 cells were transfected with transgenes encoding APP(H609TAG)-HaloTag, PylRS-AF and Pyl-tRNA$_{CUA}$, and incubated in the presence and absence of 60 µM BCNK for 45 hours. Cell lysates were analyzed with Western Blotting using rabbit anti-myc antibody **(a)** and mouse anti-APP(643-695) antibody **(b)**. A negative control is shown with untransfected (Un) cells.

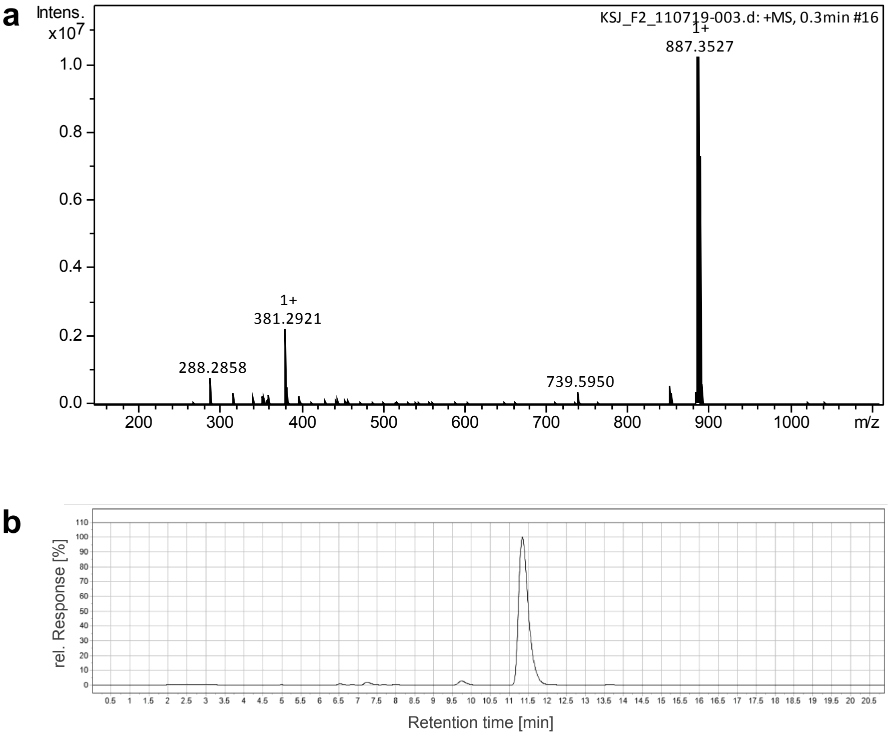

Figure S8. Mass and liquid chromatography characterizations of QSY21-chloroalkane. (a) High-resolution mass characterization of purified QSY21-chloroalkane. **(b)** HPLC chromatogram of purified QSY21-chloroalkane.

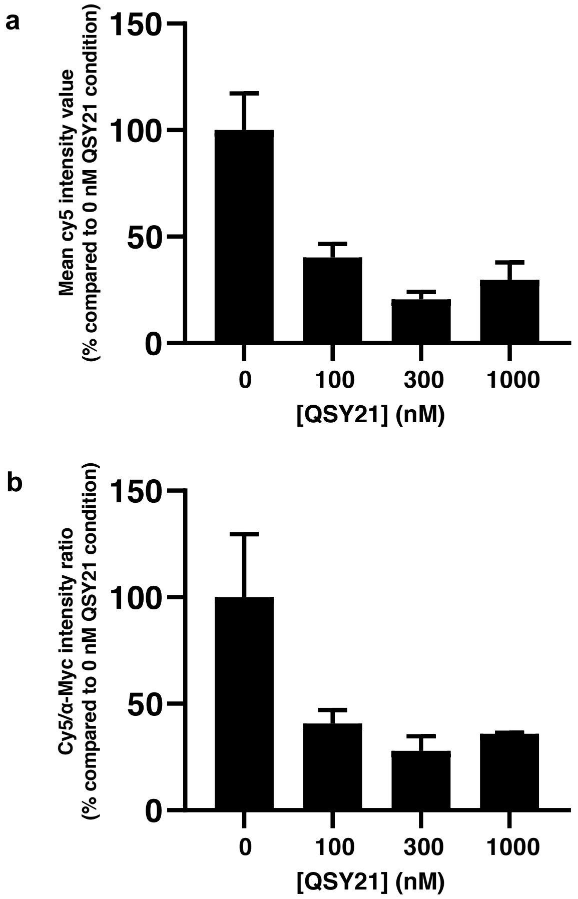

Figure S9. Quantification of FRET-based quenching efficiency of cy5 on APP(H609BCNK)-HaloTag by QSY21-chloroalkane. (**a**) cy5 intensity values (shown as percentage compared to when no QSY21-chloroalkane is represent) upon increasing concentrations of QYS21-chloroalkane. (**b**) Intensity ratio between tetrazine-cy5 and α-myc/Alexa488 (shown as percentage compared to when no QSY21-chloroalkane is present). Error bars, ± SEM. Each condition represents >12 cells analyzed.

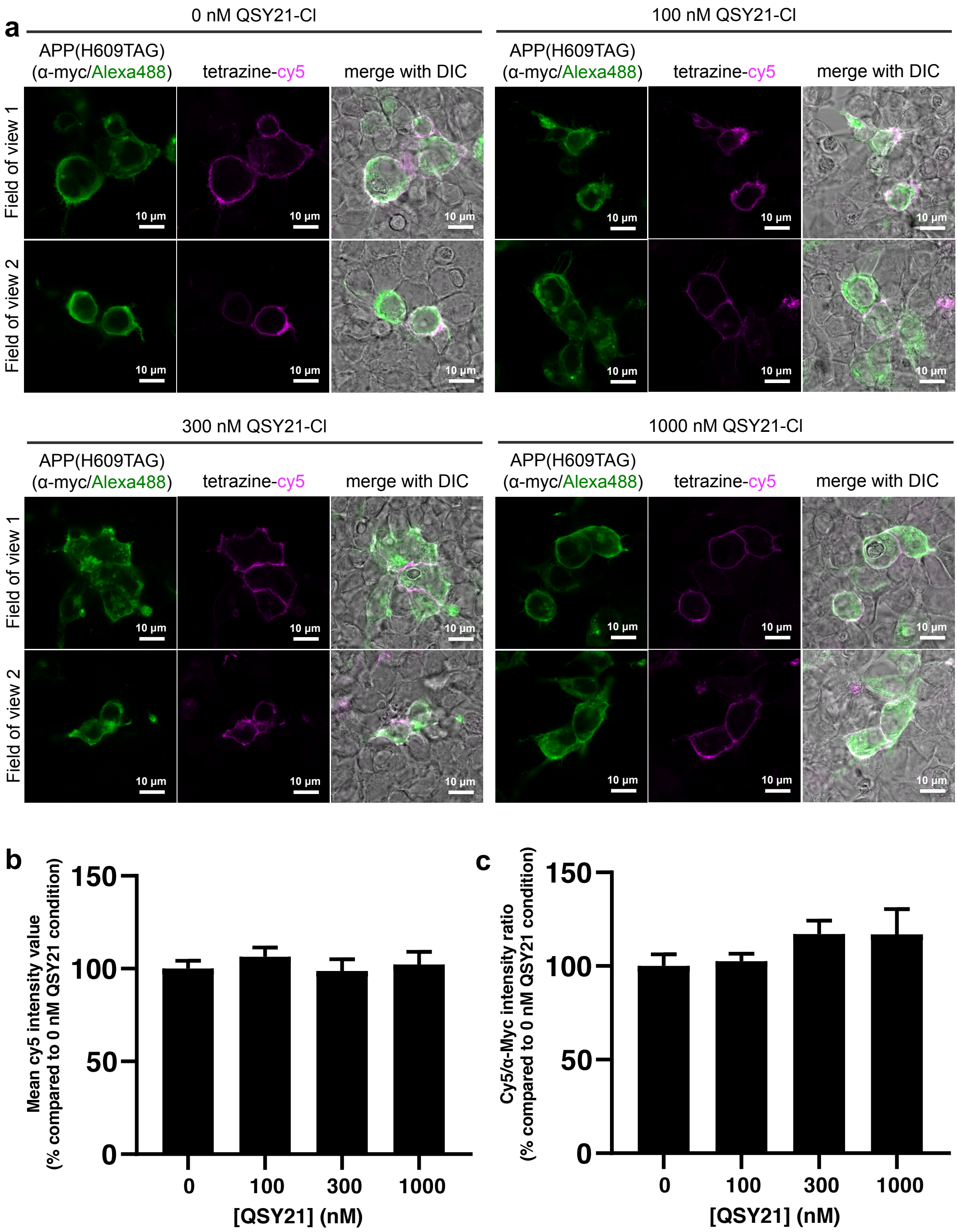

Figure S10. HaloTag on cy5-labeled APP is needed for effective quenching by QSY21-chloroalkane. (a) HEK293 cells were transfected with transgenes encoding APP(H609TAG)-myc (without HaloTag), PylRS-AF and Pyl-tRNA$_{CUA}$, and incubated with 60 µM BCNK for 45 hours. Cells were labeled live with 1.2 µM tetrazine-cy5 and indicated concentrations of QSY21-Cl at 4 °C for 30 minutes, immediately fixed, and APP-HaloTag immunostained via its C-terminal myc tag for imaging, (**b**) quantification of cy5 intensity values (shown as percentage compared to when no QSY21-chloroalkane is represent) upon increasing concentrations of QYS21-chloroalkane. (**c**) Intensity ratio between tetrazine-cy5 and α-myc/Alexa488 (shown as percentage compared to when no QSY21-chloroalkane is present). Error bars, ± SEM. Each condition represents 20 cells analyzed.

Methods

Plasmid assembly

The APP mutant constructs were cloned via site-directed mutagenesis using pEGFP-n1-APP695 plasmid (Addgene #69924) as a template. The site-directed mutagenesis was carried out by PCR amplification using mutagenic primers to place the amber stop codon (TAG (*)) at a desired position within the amyloid-β (Aβ) segment of the APP including H602*, D603*, H609*, H610* and Q611*. Subsequently, all APP mutants as well as the wild-type APP gene were subcloned into a previously reported plasmid (pPB) containing 4 copies of amber-suppressor *Methanosarcina mazei* pyrrolysyl tRNA for genetic code expansion in mammalian cells[1] using restriction cloning at the *Nhe*I and *Not*I restriction sites. To construct the APP-EGFP reporter plasmid, pEGFP-n1-APP(H609TAG) was digested with *Nhe*I and *Not*I restriction enzymes and sub-cloned into the pPB vector to generate pPB-APP(H609TAG)-EGFP_4xPylT. To construct the APP-HaloTag molecular beacon reporter plasmid, HaloTag was amplified from YIP-aga1P-HaloTag and subcloned into pPB-APP(H609TAG)-EGFP_4xPylT via restriction enzyme digest (with *Age*I and *Not*I sites to remove EGFP and replace with HaloTag) to obtain pPB_APP(H609TAG)-Halotag_4xPylT. pPB-MmPylRS-AF-4xPylT was a gift from Jason Chin.

Synthesis of QSY21-chloroalkane (QSY21-Cl)

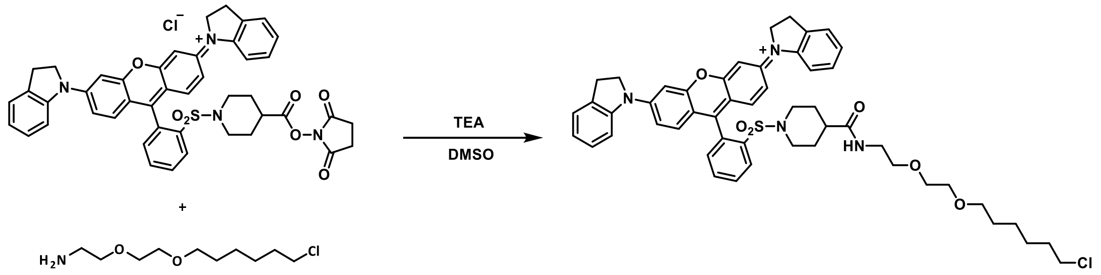

To synthesize QSY21-chloroalkane, commercially available QSY21 succinimidyl ester (1.2 mmol, 1 eq) was dissolved in dimethyl sulfoxide (DMSO) in a microcentrifuge tube. Subsequently, 2-(2-((6-

chlorohexyl)oxy)ethoxy)ethan-1-amine (1.44 mmol, 1.2 eq) and trimethylamine (12 mmol, 10 eq) were added. The reaction was shaken vertically by rotator-mixer for 5 h. The crude product was purified using UHPLC (Thermo Scientific™-Ultimate™ 3000) equipped with a Sunfire™ Prep C18 column (OBD™, 5µm, 19*150 mm) and a variable wavelength detector (Thermo Scientific VWD-3400). The gradient used was 20-80% acetonitrile in 0.1% trifluoroacetic acid in water. Solutions containing product (monitored at 230nm and 650nm) were collected and combined. Solvents were removed under reduced pressure followed by lyophilization. The product was further characterized using LC-Quadrupole-Time-of-Flight Tandem Mass Spectrometer (Compact QTOF, Bruker). MS Calculations for $C_{51}H_{56}ClN_4O_6S^+$ $[M]^+$ = 887.3604, found 887.3527.

Tissue culture

HEK293 cells (ATCC) were cultured in DMEM (Thermo Fisher) media supplemented with 10% fetal bovine serum (FBS; Thermo Fisher). Cells were passaged after reaching 80 – 90% confluence, by washing cells with PBS and trypsinization.

Live cell labeling of amyloid precursor protein and Aβ

HEK293 cells were seeded into 6-well plates (Corning) for western blot analysis or on glass coverslips placed in 24-well plates for imaging studies. The coverslips were incubated for 1 hour with 0.1 mg/mL poly-D-lysine (Sigma) prior to cell plating. Upon reaching ~ 80% confluence, the media was exchanged for Optimem (Thermo Fisher), transfection mixture of DNA (pPB_APPs : MmPylRS-AF = 9 : 1), plus optional 20 ng of organelle marker plasmids including mApple-Rab7a-7 (Addgene #54945) or mApple-TGNP-N-10 (Addgene #54954)), lipofectamine-3000 and its accessory reagent (Invitrogen). After incubation for 6 hours at 37 °C, the media was exchanged for DMEM (Thermo Fisher) supplemented with 10% FBS (Thermo Fisher), 1% Pen-strep (Thermo Fisher) and 60 uM BCNK (Sichem, catalogue no. SC-8016). Subsequently, cells were grown in presence of BCNK for 45 hours. To label the amyloid-β (Aβ) segment of APP, cells were washed twice with PBS to remove excess BCNK. Cells were labeled

in cold DMEM/FBS/Pen-strep for 30 minutes with 1.2 μM tetrazine-Cy5 (3-(p-Benzylamino)-1,2,4,5-tetrazine-Cy5), which was determined to be ≥90% pure by HPLC (Jena Bioscience, catalogue no. CLK-015-05) and subsequently washed twice with ambient-temperature PBS to remove excess tetrazine-Cy5. To allow proteolytic processing of APP, cells were incubated at 37 °C for 2 hours post-labeling in DMEM/FBS/Pen-Strep.

For the double labeling experiment on APP(H609BCNK)-HaloTag with tetrazine-Cy5 and QSY21-Cl, cells were transfected as above and labeled for 30 minutes with 1.2 μM tetrazine-Cy5 and 100-1000 nM QSY21-Cl in cold DMEM/FBS/Pen-strep and subsequently washed twice with ambient-temperature PBS to remove excess probe.

SDS-PAGE and western blot analyses of APP and Aβ

Cells were scraped in PBS and collected by centrifugation at 250 g for 10 minutes at 4 °C and subsequently lysed on ice for 30 minutes with cold RIPA buffer (Pierce) supplemented with protease inhibitor cocktail (Pierce). Lysates were clarified by centrifugation at 15,000 g for 10 minutes at 4 °C. Supernatants were collected and protein concentration normalized using BCA assay (Pierce). The lysates were resolved under the reducing condition in protein loading buffer (62.5 mM Tris pH 6.8, 2% SDS, 10% glycerol, 100 mM DTT, 0.01% bromo-phenol blue) by 12% or 15% SDS-PAGE. The gels were run at 150V for 60 minutes using Mini-PROTEAN Tetra System (Bio-Rad). Afterward, the proteins were transferred onto a nitrocellulose membrane using the Mini-PROTEAN Tetra System (Bio-Rad). The membrane was blocked with 5% skimmed milk in TBS-T (20 mM Tris, 150 mM NaCl, 0.1 % Tween 20 pH 7.6) for 1 hour at room temperature and then incubated overnight at 4 °C with primary antibodies specific for Myc epitope tag (Cell Signaling (9B11 and 71D10)), GFP (Abcam (ab290), APP (Millipore (MAB343)) or Aβ (Millipore (AB5078P)), using concentrations recommended by the manufacturers. The membrane was washed several times with TBS-T and incubated with corresponding HRP-conjugated secondary antibodies (Bio-Rad) for 1 hour at room temperature. The

membrane was washed several times with TBS-T and developed using SuperSignal West Pico Chemiluminescent Substrate (Pierce) according to the manufacturer's instructions. The signal on the membrane was photographed using ImageQuant LAS500 (GE Healthcare).

Fixed cell imaging

After labeling, the cells were fixed using 4% paraformaldehyde in PBS for 10 mins at room temperature. The fixed cells were permeabilized using cold absolute methanol and blocked using 3% bovine serum albumin (BSA) or 5% skimmed milk solution in PBS for 1 hour at room temperature. The cells were subsequently immunostained with primary antibodies α-Myc (Cell Signaling (9B11)), α-FLAG (Cell Signaling (D6W5B)) and α-Aβ (Millipore (AB5078P)) for 1 hour at room temperature, washed 3 times with PBS and immunostained with fluorescently labeled secondary antibodies (goat anti-mouse IgG H&L (Alexa Fluor®-488) (Abcam (ab150113)) or goat anti-rabbit IgG H&L (Alexa Fluor®-568) (Abcam (ab175471))). Cells were stained with DAPI (Sigma) per manufacturer’s protocol. Cells were imaged using Zeiss LSM800, or Olympus Fluoview FV3000 confocal microscopes with appropriate laser and filter settings.

In-gel cy5 fluorescence visualization of APP and Aβ

HEK293 cells expressing APP(H609BCNK)-α-myc, APP(H609BCNK)-EGFP or APP(H609BCNK)-HaloTag were labeled for 30 minutes with 1.2 μM tetrazine-cy5 in cold DMEM/FBS/Pen-strep and subsequently incubated at 37 °C for 2 hours post-labeling in order to allow proteolytic processing of APP as described in the previous section. Cells were lysed on ice for 20 minutes and the lysates were cleared by centrifugation at 20,000 g at 4 °C. The lysates were collected and protein concentration was normalized using BCA assay (Thermo Fisher). Afterwards, 20 μg protein from lysate was analyzed on 15% SDS-PAGE gels, fixed in buffer containing 30% ethanol and 10% acetic acid, and subsequently imaged using ChemiDoc™ MP Imaging System (BIO-RAD) with cy5 laser and filter setup.

Reference

[1] W. H. Schmied, S. J. Elsässer, C. Uttamapinant, J. W. Chin, *Journal of the American Chemical Society* **2014**, *136*, 15577-15583.
